# The influence of musicality on expressive fable reading: adult- and child-directed perspectives

**DOI:** 10.3389/fpsyg.2026.1720324

**Published:** 2026-03-16

**Authors:** Markus Christiner, Christine Groß, Valdis Bernhofs, Ana Nezmah, Katharina Korecky-Kröll

**Affiliations:** 1 Music Psychology and Brain Research Section, Department of Psychology, University of Graz, Graz, Austria; 2 Jazeps Vitols Latvian Academy of Music, Riga, Latvia; 3 Department of Child and Adolescent Psychiatry, Saarland University, Homburg, Germany; 4 School of Health, Education, and Social Sciences, SRH University Heidelberg, Heidelberg, Germany; 5 Theatre Faculty of the Academy of Performing Arts in Prague, Prague, Czechia; 6 Austrian Centre for Digital Humanities, Austrian Academy of Sciences, Vienna, Austria; 7 Department of German Studies, University of Vienna, Vienna, Austria

**Keywords:** fable reading, melody, musical ability, musical training, reading, singing, singing behavior during childhood, expressive reading

## Abstract

Reading ability within the field of musicality research is multifaceted and has been examined from various perspectives to elucidate the nature and basis of its relationship with musical capacities. In this study, we specifically address expressive reading ability—a dimension that has received relatively little detailed investigation to date—within the broader context of musicality. This research aimed to identify which musical capacities are associated with expressive reading abilities and to examine whether these associations vary depending on whether the intended audience is adults or children. We recruited 67 participants who were assessed on their expressive reading ability by having them read the well-known fable “The North Wind and the Sun.” Additionally, participants completed a series of musicality assessments and questionnaires. Results revealed that both adult-directed speech (ADS) and child-directed speech (CDS) were associated with melodic abilities and singing behavior during childhood. Although CDS was also found to be linked with musical engagement (such as training) and rhythmic ability, regression analyses indicated that melodic skills were the strongest predictors for both CDS and ADS. The findings underscore the significant predictive role of melodic competence in engaging fable presentation. Melodic competence correlated with rater-assessed listener engagement, potentially reflecting shared underlying processes such as attention, tension, and memory.

## 1 Introduction

Reading is one of the most fundamental literacy skills, to attentively process written texts by comprehending, interpreting, and critically engaging with the information presented. Therefore, understanding the underlying mechanisms that support and foster reading ability has become a topic of growing research interest. A number of studies have documented associations between reading ability and musicality—a multifaceted skill whose relevance to reading, while not immediately apparent, has increasingly drawn scholarly attention (Dellatolas et al., 2009; Eccles et al., 2021; Gordon et al., 2015; Partanen et al., 2022; Seither-Preisler et al., 2014).

Musical abilities have been increasingly recognized as important contributors to various cognitive and linguistic skills. Studies have provided evidence that several core musical competencies—such as singing, rhythmic skills, and melodic abilities—are closely linked to language abilities including speech perception (Besson et al., 2011; Chobert et al., 2014; Christiner, 2020; Groß et al., 2023; Moreno et al., 2009), phonetic ability (Christiner et al., 2023; Milovanov, 2009; Milovanov and Tervaniemi, 2011), phonological awareness (Christiner et al., 2022c; Degé and Schwarzer, 2011; Eccles et al., 2021; Gordon et al., 2015), and pronunciation as well as mimicking skills (Christiner et al., 2021; Christiner et al., 2022b; Coumel et al., 2023). These language-related skills are also fundamental in accounting for individual differences in reading proficiency and reading performance in both first and second language contexts.

Studies on reading ability and musicality are diverse and address multiple dimensions. Consequently, findings regarding the overlap between reading ability and musicality are mixed; while the majority of studies report a significant association (Anvari et al., 2002; Eccles et al., 2021; Fonseca-Mora et al., 2021; Gomez-Dominguez et al., 2019; Partanen et al., 2022; Seither-Preisler et al., 2014), others do not, or suggest that the observed relationship may be explained by other underlying cognitive abilities (Banai and Ahissar, 2013; Swaminathan et al., 2018).

Most studies on the influence of musicality on reading ability have predominantly focused on children or second language learners, populations in which reading is an active component of the learning process (Gordon et al., 2015). Research has reported positive associations between reading ability and both tonal and rhythmic musical skills, indicating that multiple aspects of musicality contribute to the development of reading proficiency. Among these, evidence has been provided that active musical training is associated with enhanced auditory processing and attention mechanisms, which positively correlate with reading fluency and spelling skills in children (Seither-Preisler et al., 2014). Another study on children suggested that musical perceptual skills are linked to reading ability regardless of musical training (Partanen et al., 2022). Research has also shown that musical perception remains a significant predictor of reading ability even when other crucial cognitive abilities such as math, digit span, and vocabulary are included in statistical models (Anvari et al., 2002). In the context of foreign language learning, studies with both children and adults have identified associations between musicality and foreign language reading skills (Fonseca-Mora et al., 2021; Gomez-Dominguez et al., 2019). However, research also reported the opposite. Although musical training correlates with reading ability, this relationship diminishes when controlling for general cognitive abilities (Swaminathan et al., 2018). The study concludes that the connection between musicality and reading ability may be largely driven by broader cognitive factors. Similar conclusions have been reached by other research suggesting that in general the relationship between musicality and language ability may result from pre-existing cognitive and perceptual abilities underlying both domains (Swaminathan and Glenn Schellenberg, 2020).

The studies discussed above reveal considerable variation not only in the musical measures (musical training, musical ability and performance variables) but particularly in the methods used to assess reading skills. For instance, Seither-Preisler et al. (2014) tested reading speed and fluency to assess reading skills. Partanen et al. (2022) employed the ALLU-T4A and ALLU-T5B tests (Lindeman, 1998) which assess auditory language comprehension at both basic and more complex linguistic levels. Gomez-Dominguez et al. (2019) assessed reading by decoding familiar and unfamiliar words through letter-sound correspondence, while Fonseca-Mora et al. (2021) examined reading fluency and silent reading ability in adults learning a foreign language. Anvari et al. (2002) used the reading subtest of the WRAT-3 which involves participants first recognizing and naming letters, followed by reading aloud a series of increasingly difficult words presented in isolation. In contrast, the study by Swaminathan et al. (2018) used the Nelson-Denny Reading Test, measuring reading comprehension through multiple-choice questions that evaluate both literal and inferential understanding, as well as reading rate (Brown et al., 1993).

Reflecting the diversity of the previously discussed measures, reading ability in the context of musicality studies is typically evaluated using tests that assess phonological awareness, spelling, reading comprehension, reading fluency, and reading speed, all aimed at identifying ways to assess individual differences in reading ability. However, inconsistencies in the association between reading ability and musicality may be attributed to both variability in musicality measures and substantial differences in the reading tasks used (Eccles et al., 2021; Gordon et al., 2015). Additionally, participant age and whether the reading task was conducted in the native or a foreign language represent further factors influencing the relationship between musicality and reading ability. Accurately delineating the reading tasks employed, alongside relevant participant characteristics such as age and target audience, is fundamental for validly identifying the specific reading processes under investigation.

When adults read a story or fable for a target audience, this reading task differs significantly from the previously mentioned reading tasks employed by other researchers and could be characterized as expressive (Paige et al., 2012), or prosodic reading performance (Wade-Woolley et al., 2022). A skilled reader does not solely prioritize reading accuracy or reading speed, but also employs prosodic features—such as melody and rhythm—to segment text into meaningful units (Bailly et al., 2022; Binder et al., 2013; Dowhower, 1991). By modulating pitch and varying intensity throughout passages, the skilled reader not only seeks to capture and maintain the audience’s attention (Bailly et al., 2022), and enhance comprehension (Schwanenflugel et al., 2004), but also create tension (Larrouy-Maestri et al., 2025). Furthermore, research has demonstrated that melody recognition in speech can enhance memory for spoken utterances (Christiner et al., 2021; Christiner et al., 2023). Consequently, oral reading—particularly expressive reading—shares fundamental elements with musical performance, highlighting the important role musical skills may play in modulating reading abilities. Despite these similarities, the relationship between expressive reading and musicality has received limited attention and remains insufficiently explored.

If expressive reading performance is employed in studies on the overlap between musicality and reading ability, three aspects are of particular interest: First, reading a fable aloud closely resembles a musical performance, as it involves carefully shaping prosodic, rhythmic, and melodic parameters to capture and maintain the attention of the audience. Therefore, it is reasonable to hypothesize that individuals with higher musical skills are more likely to deliver a more effective and expressive reading performance. In the same vein, it is crucial to investigate which specific musical variables play a significant role in explaining individual differences in expressive reading, thereby addressing the second crucial aspect. It must also be acknowledged that studies on musicality and reading ability employ a wide array of musicality measures—including musical ability, musical training, singing, and listening skills. Musicality encompasses diverse skills that relate differently to language abilities; while some musical skills, such as singing behavior in childhood or singing ability, show strong links to oral language performance, others may contribute in more specific or limited ways depending on the language function assessed (Christiner et al., 2022a; Christiner and Reiterer, 2015). Therefore, assessing musicality from multiple dimensions is essential to determine which particular facets most strongly contribute to expressive reading performance. Third, to disentangle domain-general auditory-motor mechanisms from music-specific skills, phonological and prosodic measures known to correlate with musicality should be included as competing predictors (i.e., to test whether they outperform established musicality measures). Prior research reported links (e.g., Christiner and Groß, 2025) between auditory phonological pattern recognition and musicality, as well as between speech imitation/prosodic imitation/accent faking and singing ability (e.g., Christiner et al., 2022a; Christiner et al., 2022b; Christiner et al., 2022c; Coumel et al., 2023). These language measures target similar underlying mechanisms as music perception measures and singing measures: auditory phonological pattern recognition (multi-syllabic sequences requiring differentiation and retention, mirroring musical sequences) and speech imitation (somatosensory prosodic reproduction, paralleling singing).

Fourth, the reading performance must be deliberately adjusted to suit the target audience in order to maintain engagement and convey the narrative effectively. Building on the well-established links between musical abilities and language skills, another aspect of high interest lies in the overlap between expressive reading performance and musical ability: The distinction between adult-directed speech (ADS) and child-directed speech (CDS) is particularly relevant. In contrast to ADS, CDS is characterized by higher pitch, greater pitch variability, exaggerated intonation, and other enhanced musical features, which are thought to facilitate attention (Fernald et al., 1989; McMullen and Saffran, 2004; Soderstrom et al., 2008). In addition, CDS shows more even-timed rhythmic patterns than ADS (i.e., a more pronounced syllable-timed quality), even in stress-timed languages (e.g., Payne et al., 2015). Although CDS shows much variation depending on the language, the culture, the child’s age, the adult’s gender and as well as individual preferences (for an overview on potentially relevant social and linguistic variables see, e.g., Soderstrom and Bortfeld, 2020) caretakers, in general, tend to produce more acoustically extreme language features (Kuhl et al., 1997) and speech targeted at children show more pitch variations and carries longer pauses aiming to convey the message more precisely (McMullen and Saffran, 2004). Consequently, language targeted at children has often been described as more musical in its nature (Murphey, 1990). This suggests that language targeted at children exhibits stronger overlaps between musicality and language abilities than language directed at an adult audience.

This study seeks to bridge an existing research gap by exploring the relationship between musicality and expressive reading abilities. Consequently, to systematically explore the intersection of musicality and reading performance, we formulated three central research questions that encompass expressive reading domain. First, we sought to examine whether, and if so which, musical variables are associated with expressive reading ability when reading the fable “The North Wind and the Sun.” Second, we aimed to examine whether specific musical variables were most strongly associated with rater-assessed fable engagement across musicality dimensions (melodic, rhythmic, singing behavior, musical engagement, passive listening, perceived musical relevance). Additionally, we wanted to examine whether prosodic imitation, auditory phonological skills, and self-efficacy were associated with engaging fable presentation, as prosodic and auditory skills demand similar cognitive abilities as musical processing (e.g., attention, temporal processing). Third, we examined whether the same musical capacities are associated with expressive reading of fables for ADS versus CDS, given CDS’s typically more musical nature.

## 2 Materials and methods

### 2.1 Participants

A sample of 67 participants was recruited for this study, all of whom provided informed consent and participated voluntarily. The cohort comprised 46 female, 20 male, and 1 non-binary individual. Regarding musical training, 28 participants reported no prior instrumental acquisition, whereas 39 indicated having learned at least one musical instrument during their lifetime. The native language of all participants was German. The mean age was 35.4 years (*SD* = 13.1). Recruitment occurred via online platforms and university channels, with inclusion criteria specifying a minimum age of 18 years. The study was conducted according to the guidelines of the 567 Declaration of Helsinki, and approved by the Institutional Review Board of the University of Graz 568 GZ. 39/106/63 ex 2024/25.

The participants’ educational status was entered according to the educational status which was completed at testing time. The educational background is as follows: 14 participants completed technical and vocational school, 16 attended secondary academic school (general qualification for university entrance), 9 completed post-secondary non-tertiary education, 11 undertook bachelor studies, and 17 held a master’s degree or doctoral degree.

### 2.2 Reading the fable North Wind and the Sun

To assess individual differences in reading ability, we employed a reading task in which participants were asked to read the German version of the well-known fable “The North Wind and the Sun” twice. This fable has been frequently used as reading-aloud material in studies investigating different phonetic or phonological characteristics (e.g., Carlsen et al., 2020) of different languages (e.g., Hove, 2014) different varieties of a language (e.g., Hahn and Siebenhaar, 2019 on German), different communicative settings (e.g., Künzel, 2001) or different groups of speakers (e.g., Schuster et al., 2006; Hönig et al., 2020). For the first reading, participants were instructed to read the text as accurately as possible, imagining their intended audience to be adults. They were encouraged to read the fable to the best of their ability and to engage the listeners’ attention.

In the second reading, participants were again asked to read the same fable; however, this time they were instructed to read it as if their target audience were children, aiming to read in a manner that would capture and maintain the attention of young listeners.

The reading task used in this study serves multiple functions. First and foremost, it requires participants to engage and captivate their listeners, emphasizing the importance of expressive and dynamic performance. This entails not only accurate reading but also effective modulation of prosody, rhythm, and intonation to maintain listener attention. Additionally, the reader adapts their performance depending on the target audience—whether adults or children—highlighting the role of audience-specific speech styles in communication. Thus, the task integrates both decoding skills and communicative performance, underscoring the interplay between linguistic and musical abilities.

To evaluate the performances of both reading tasks, we employed established methods and recruited raters who had to fulfil two main criteria. Raters were required to be German native speakers with university education in linguistics. For the ratings, recordings were uploaded to an online platform. Raters were instructed to evaluate how engaging the fable presentation was, specifically assessing the extent to which it captured and sustained the listener’s attention throughout the entire narrative. To facilitate accurate evaluation, raters were also provided with the original instructions given to the participants.

To assess the reliability of the ratings, intraclass correlation coefficients (ICCs) were calculated. Following Koo and Li (2016), a minimum of 3 raters per task ensures reliable ICC estimates; larger numbers enhance score precision and data robustness. We therefore aimed to keep rater numbers well above this recommendation across tasks requiring ratings (singing ‘Happy Birthday’ and prosodic imitation). Fifteen raters were invited and participated in the evaluation; however, only 14 rated both the ADS and CDS fable readings, while one rater provided ratings solely for the adult-directed fable reading. Inter-rater reliability was above the threshold of 0.70 for ADS (*α* = 0.84) and CDS (α = 0.73) (see Supplementary Tables S1, S2).

### 2.3 Musicality measures

To assess the auditory musical abilities of the participants, we used four different musicality measures, each targeting core competences in rhythmic processing, auditory scene analysis, melodic discrimination, and intonational sensitivity.

#### 2.3.1 Computerized adaptive beat alignment test (CA-BAT)

The CA-BAT is a modern psychometric instrument for assessing beat perception, developed by Harrison and Müllensiefen (2018). It builds on the classical Beat Alignment Test (BAT), adopting a two-alternative forced-choice (2-AFC) paradigm wherein participants hear two versions of a musical excerpt, each overlaid with a beep track. One version is metrically aligned (target), while the other is systematically misaligned (lure). The task is to identify the version that is better aligned with the underlying musical beat.

The CA-BAT integrates key innovations from contemporary psychometrics: (1) Item Response Theory (IRT) ensures comparability of ability estimates across different item sets; (2) Computerized Adaptive Testing (CAT) selects maximally informative items based on the participant’s estimated ability; and (3) Automatic Item Generation enables the creation of large-scale item banks with controlled item difficulty, derived from empirically validated parameters such as beat-track displacement and musical track characteristics.

Test items are derived from a pool of 32 five-second musical excerpts from diverse genres, with canonical beat locations empirically determined through drummer performance. Difficulty is primarily modulated by the degree and direction of beep-track displacement. The test dynamically adapts item selection to the participant’s performance, enhancing precision and reducing test length without compromising reliability.

Validation across multiple studies demonstrated high construct validity, significant correlations with musical training (*r* ≈ 0.40), and good test–retest reliability (*r* = 0.67) in controlled laboratory settings. However, performance reliability decreases markedly under uncontrolled online conditions, emphasizing the importance of standardized administration. The CA-BAT is particularly suited for research on individual differences in beat perception and its neural and behavioral correlates.

#### 2.3.2 Melodic discrimination test (MDT)

Melodic discrimination ability was assessed using the Melodic Discrimination Test (MDT), developed by Harrison et al. (2017). The MDT is a computerized adaptive test based on modern psychometric principles, including item response theory (IRT), automated item generation (AIG), and computerized adaptive testing (CAT). It is designed to provide an efficient and reliable measure of an individual’s ability to detect local changes in melodic structure.

In each trial, participants are presented with three melodic sequences: two foils and one target. The foils are identical except for a transposition of one semitone up or down, while the target melody contains a single altered pitch that disrupts the underlying melodic contour. Participants are asked to identify the deviant melody using a three-alternative forced-choice (3-AFC) format.

Melodies are algorithmically generated from a statistical model trained on a corpus of Irish folk songs. Each melody consists of 3–16 notes, and test items vary systematically in terms of difficulty, which is estimated based on musical-structural features such as contour violations and tonal regularity. Transpositions are used to control for absolute pitch cues, ensuring that performance reflects relative pitch processing.

The test is delivered online through the psychTestR platform, and responses are scored adaptively. The MDT demonstrates high internal consistency (Cronbach’s *α* > 0.8) and construct validity, with strong associations to musical training and related auditory abilities.

#### 2.3.3 Mistuning perception test (MPT)

To assess listeners’ sensitivity to vocal mistuning, we employed the Mistuning Perception Test (MPT), a newly developed adaptive and ecologically valid measure introduced by Larrouy-Maestri et al. (2019), test uses short (6–12 s) audio excerpts drawn from commercial pop recordings (Bittner et al., 2014), in which the vocal track is either left unaltered or artificially pitch-shifted relative to the instrumental accompaniment.

In each trial, participants perform a two-alternative forced-choice (2AFC) task, hearing both the original and the pitch-shifted version of the same musical excerpt in random order. Their task is to identify the mistuned version. In the initial development phase, 333 listeners completed the test in a fixed-format version to allow for explanatory item response modeling, which enabled the calibration of an adaptive version of the test.

The final adaptive implementation dynamically adjusts item difficulty based on participant responses and was validated in a subsequent experiment with 66 individuals representing a wide range of musical backgrounds. Results demonstrated robust test–retest reliability as well as strong convergent and divergent validity. The MPT is suitable for both experimental and applied contexts, offering an efficient and ecologically valid method to quantify mistuning perception ability.

#### 2.3.4 Musical scene analysis test

Auditory scene analysis ability in a musical context was assessed using the Musical Scene Analysis (MSA) Test, an adaptive, ecologically valid instrument developed by Hake and co-authors (Hake et al., 2024). The MSA Test presents participants with a target instrumental excerpt followed by a short polyphonic musical mixture and requires a two-alternative forced-choice (2AFC) judgment: participants must decide whether the target instrument is present in the mixture.

Stimuli are drawn from realistic multi-track recordings and systematically manipulated across three key parameters: the target-to-mixture ratio (TMR), the number of instruments in the mixture, and the instrument type. The test employs a Bayesian adaptive algorithm (PsyBayes) to estimate participants’ detection thresholds with high efficiency and precision.

Validation studies across both normal-hearing and hearing-impaired populations demonstrated good test–retest reliability, sensitivity to hearing status, and convergent validity with other auditory and musical tasks. The MSA Test is freely available, time-efficient, and particularly suitable for examining the perceptual effects of hearing impairment in complex musical contexts.

### 2.4 Singing ability

For assessing individual differences in singing ability, we used the “Happy Birthday” song singing task, which is suitable for both professional and non-professional singers (Bella et al., 2009; Bella et al., 2007). Participants were instructed to sing a short passage of “Happy Birthday” as accurately as possible in their own singing voice. This task has been used in multiple studies and has proven to be highly effective for assessing individual differences in singing ability (Christiner et al., 2022a; Christiner and Reiterer, 2013; Christiner et al., 2022b).

The participants’ singing performances were reviewed, trimmed to remove extended pre-singing silence for rater comfort, and had their loudness adjusted before being uploaded to a rating platform. Subsequently, they were rated and evaluated by nine professional musicians, who assessed four criteria: melodic accuracy, rhythmic accuracy, vocal range, and voice quality (rater numbers and recruitment rationale explained in Section 2.2). These criteria were combined into a composite score, as this approach has been employed in several previous studies (Christiner et al., 2022a; Christiner and Reiterer, 2013; Christiner and Reiterer, 2019; Christiner et al., 2022b). Inter-rater reliability was calculated using intra-class correlation coefficients, which indicated that the ratings were reliable, with ICC values ranging from 0.70 to 0.88 (see Supplementary Section 2).

### 2.5 Musical engagement score

We also collected information on participants’ active musical engagement, aiming to balance self-reported singing and instrumental activities. They were asked to indicate the average number of hours per week they spent singing and playing a musical instrument. Participants were instructed to consider a retrospective period of the past 3 years when estimating these hours. Additionally, participants reported the number of instruments they play, which served as an additional indicator of their musical involvement.

All three variables were log-transformed and subsequently standardized (z-transformed) prior to aggregation into a composite musical engagement score. Inspection of the intercorrelations revealed a strong positive association between the number of instruments played and the hours spent playing a musical instrument (*r* = 0.65). Accordingly, these two variables were combined into a single score. The final musical engagement variable (Musical Engagement Score) was computed by summing this combined score and the standardized singing hours per week.

### 2.6 Scales for singing behavior during childhood, music relevance, and passive music listening habits

To comprehensively assess participants’ musical behaviors and subjective valuation, three core conceptual domains were measured using dedicated multi-item scales: (1) singing behavior during childhood, (2) perceived relevance of music, and (3) passive music listening habits. All items across these scales were rated on an 11-point Likert scale ranging from 0 (strongly disagree/not at all) to 10 (strongly agree/completely).

Singing behavior during childhood was evaluated with a previously validated five-item scale. In language research, acquiring a language during childhood is considered crucial, as this factor is one of the most important predictors of achieving high proficiency. Similarly, early music training in childhood is essential and facilitates the development of musical expertise later in life. As a counterpart to linguistic measures, we used a previously developed multi-item scale that assesses singing behavior during childhood. This construct also serves to capture informal musical engagement among individuals without formal training.

Perceived relevance of music describes participants’ subjective importance and personal valuation of music, assessed via a newly developed five-item scale.

Passive Music Listening Habits represents a widespread, everyday form of musical engagement. A four-item scale was employed to capture this behavior, from which a composite Passive Music Listening Habits score was derived.

Reliability analyses confirmed satisfactory internal consistency for all three constructs (Cronbach’s *α* ranging from 0.74 to 0.92). Further details on the individual items and rating procedures are provided in Supplementary Section 3.

### 2.7 Prosodic imitation tasks

We used language stimuli that were already used in previous research (Christiner et al., 2021; Christiner et al., 2023; Christiner et al., 2022a; Christiner et al., 2022b; Christiner et al., 2022c). For the prosodic imitation tasks, stimuli consisting of nine and eleven syllables—spoken by three male and three female native speakers—were created for each of the selected languages: six in Mandarin and six in Tagalog. Both languages are rather unfamiliar to participants, ensuring performances primarily reflect prosodic imitation ability rather than language contact or semantic knowledge.

The participants were asked to listen to the language samples three times, each time separated by a pause of 50 ms, before they had to repeat the language phrases as prosodically accurately as possible. This method of assessing foreign language capacity reveals how well and how quickly individuals can acquire unfamiliar prosodic patterns in settings resembling early language learning environments without established semantic representations.

Before the main task, participants completed a familiarization task to ensure they understood the procedure. During this phase, Tagalog and Mandarin samples that were different from those used in the main study were played to acquaint them with the process. After the familiarization phase, participants imitated six Tagalog and six Mandarin samples. The sequence of the samples was always randomized for each participant. The recordings of the participants were then processed for the follow-up analysis, during which native speakers assessed their performances. The recordings were cut, checked, and their loudness was normalized for this purpose.

To evaluate the prosodic performances, we recruited native speakers (16 Tagalog, 40 Mandarin) to assess individual differences in language production (rater numbers and recruitment rationale explained in Section 2.2). The recordings of the participants were uploaded to an online platform for this purpose. Raters used a scale from 0 (“min”) to 10 (“max”) and were instructed to evaluate the overall prosodic performance globally for each language stimulus in the corresponding language – specifically assessing how well participants imitated the prosodic contour (i.e., intonation/speech melody, rhythm, and stress patterns). They received instructions noting that utterances “may not be understood clearly” or “may not be meaningful” due to unfamiliarity with Mandarin/Tagalog. Each rater assessed all samples in their respective language within a 14-day time window. Our custom platform divided ratings into runs of 30–40 samples, each with 3 randomized practice trials (poor/average/good; excluded) for calibration. Raters could pause/resume anytime. To assess the reliability of the ratings, intraclass correlation coefficients (ICCs) were computed. The results showed high interrater reliability (ICCs ranging from 0.90–0.98; full details in Supplementary Tables S7–S18). Since language typology plays a subordinate role in prosodic imitation (Christiner, 2020; Christiner et al., 2023), we computed a composite prosody score across the 12 items in this sample (Cronbach’s *α* = 0.88).

### 2.8 Auditory phonological pattern recognition

A short version of the auditory phonological pattern recognition task (Christiner and Groß, 2025) assessed participants’ ability to detect, segment, and match unfamiliar multi-syllabic sequences based on phonological and prosodic patterns. The measure was recorded by six native speakers per language (three male, three female) and developed across 36 languages using *The North Wind and the Sun* fable. For native German-speaking participants, we selected Tagalog and Mandarin—two particularly unfamiliar languages—ensuring little or no prior familiarity while providing natural phonetic and prosodic variation.

The trials consist of words and short phrases ranging from three to eleven syllables, normalized for loudness to ensure comparability across recordings. Each trial included two components: a string and a response stimulus. The string comprised eight segments for each participant—four segments contained between three and six syllables, and four contained between seven and eleven syllables—sampling a range of phonological complexity and prosodic length. Similar variants with ten elements (five short and five long segments) or twelve elements (six short and six long segments) were also used to cover the spectrum of phonological and prosodic variation. Segments within the string were separated by brief silent intervals of 50 milliseconds. Following the string, a two-second delay, indicated by a change in screen color, preceded the presentation of the response stimulus. Response stimuli comprised 1–3 units that participants matched to the preceding string. A response was scored correct only if all units appeared somewhere in the string, irrespective of order.

For multi-unit responses, correctness required that all units appeared anywhere within the reference string, regardless of their original sequence or position. For example, in an 8-segment string trial, a 3-unit response stimulus (e.g., Segments A–B–C) was scored correct if A, B, and C were present in the string, irrespective of order. All stimuli within each trial pair originated from the same native speaker to preserve consistent prosodic cues. An example of an 8-segment trial, including possible response options, is presented in Table 1 below.

**Table 1:** Shows an 8-segment string trial.

#	String segments	Syllables	✓ Response A (correct: 1 + 3)	✗ Response B (incorrect: 3 + fake)
1	tāmen jiù juédìng	5	✓	
2	nàgèrén chuānzhè yījiàn dǒupéng	9		
3	shéi kěyǐ ràng lùrén	6		✓
4	yúshì běifēng jiù	5		
5	méi xiǎng dào, tā chuīdé yù lìhài	9		
6	dǒupéng bāojǐn zìjǐ	6	✓	
7	yúshì, běifēng zhǐhǎo rènshū	8		
8	jiēzhe, tàiyáng chūlái wēnnuǎndi	9		
-	zhàoyàole yīxià (Fake)			✗ not contained

Illustrates a possible 8-segment Mandarin trial (as they will be randomly generated) where Response A (Segments 1 + 6) is correct as both appear in the reference string, while Response B (Segment 3 + “zhàoyàole yīxià”) is incorrect because “zhàoyàole yīxià” is not contained anywhere in the string. An answer is correct only if both segments are present in the reference string.

Participants completed 12 binary trials per language, randomly sampled from 8-, 10-, and 12-segment strings (balanced “match”/"non-match” stimuli across lengths). Mean accuracy was 8.55/12 (71.3%), indicating moderate task difficulty. Large-scale validations show normal distributions (*N* = 434 + 500; Christiner, 2020; Christiner and Groß, 2025), while the NWS subsample exhibits mild ceiling effects with non-normal distribution (Shapiro–Wilk *p* = 0.006). A sample of the extracted words and phrases from one speaker is provided in Supplemental Section 6.

### 2.9 Language self-efficacy

Language self-efficacy measured domain-specific self-efficacy for language tasks directly assessed in the study (listening, imitation, expressive reading), following Bandura (2006). Language self-efficacy was assessed immediately following task exposure, capitalizing on participants’ heightened task sensitivity induced by the preceding standardized stimuli (reading, imitation, pattern recognition). This stimulus-primed assessment should enhance ecological validity by anchoring self-efficacy judgments to concrete, recent performance experiences. The 7-item scale was administered post-testing on a visual analogue scale (0 = strongly disagree to 10 = strongly agree). Internal consistency: Cronbach’s *α* = 0.74 (full item details: Supplementary material 4).

### 2.10 Statistical analysis

The statistical analyses were conducted in a series of consecutive steps, each targeting a distinct research aim. Sample size (*N* = 67 after minimal attrition <5%) supported exploratory screening of 13 predictors post-hoc (99% power at observed large effects *f*^2^ = 0.33–0.35; cf. music-language *r* = 0.44–0.71, Picciotti et al., 2018; Fonseca-Mora et al., 2021). First, correlation coefficients were calculated to explore the relationships between reading tasks and musicality measures.

Based on the inspection of the correlation matrix, regression analyses were subsequently performed. Due to substantial intercorrelations among the melodic variables (MDT, MPT, and singing), sensitivity analyses were conducted by alternately excluding each variable from regression models predicting reading abilities in both children and adults. These analyses consistently showed that one of the three melodic measures remained a significant predictor, suggesting that they reflect the same underlying melodic competence.

To address multicollinearity and enhance model stability, the three variables were combined into a single composite score representing shared melodic ability. This composite score captures the common variance of melodic perception and production, resulting in a more parsimonious and interpretable model without compromising predictive power. Exploratory stepwise regressions confirmed melodic composite score as the dominant predictor among correlated variables (see Supplementary Section 7). Confirmatory multiple regression models using the forced-entry method—including all correlated predictors—were then fitted. Two separate regression models were performed for the Nord Wind and the Sun reading (ADS and CDS) as the dependent variables.

## 3 Results

### 3.1 Descriptives

The following descriptions define the acronyms used for the variables presented in the descriptive tables. NWS refers to the “North Wind and the Sun” reading task. “Adult-directed” indicates that participants were instructed to read the fable as if addressing an adult audience, whereas “child-directed” refers to readings intended for a child audience. BAT stands for the Computerized Adaptive Beat Alignment Test (CA-BAT), MDT for the Melodic Discrimination Test, MPT for the Mistuning Perception Test, and MSA for the Musical Scene Analysis Test; all of these belong to the LongGold test battery. “Singing Happy Birthday” represents the singing performance score, while the “Musical Engagement Score” denotes the cumulative hours of instrumental practice, singing training, and instrument playing. Finally, mean scores are provided for the three multi-item scale concepts: “Singing Behavior During Childhood,” “Passive Music Listening Habits,” and “Music Relevance.”

### 3.2 Correlational analyses

To assess the associations between the two NWS reading tasks and the musicality variables, correlational analyses were performed. Correlation analyses were performed on the following variables: Auditory Phonological Pattern Recognition, Prosodic Imitation score, Language self-efficacy, objective musical perception tests (CA-BAT, MDT, MPT, MSA), the singing task “Happy Birthday,” the Musical Engagement Score, and the multi-item scales assessing Singing Behavior During Childhood, Passive Music Listening Habits, and Musical Relevance. We applied the Benjamini-Hochberg procedure to control for the false discovery rate arising from multiple comparisons. After applying the Benjamini-Hochberg correction to control the false discovery rate, all variables previously identified as significant remained significant, underscoring the robustness of our findings against multiple testing. The correlation results for the North Wind and the Sun (NWS) ADS and CDS reading tasks are presented separately in Tables 2, 3.

**Table 2:** Presents descriptive statistics (means, standard deviations) and measurement scales for all variables included in the analysis.

Variables	Mean (*M*)	Standard deviation (*SD*)	Scale
NWS ADS	5.10	1.15	0–10
NWS CDS	5.80	0.97	0–10
BAT	−0.45	1.16	*z*-scores (*M* = 0, SD = 1)
MPT	0.08	0.91	*z*-scores (*M* = 0, SD = 1)
MDT	−0.33	1.01	*z*-scores (*M* = 0, SD = 1)
MSA	0.02	0.65	*z*-scores (*M* = 0, SD = 1)
Singing happy birthday	5.54	1.60	0–10
Singing behavior during childhood	5.06	2.70	0–10
Musical engagement score	−0.15	0.71	*z*-scores (*M* = 0, SD = 1)
Passive music listening habits	4.15	2.73	0–10
Music relevance	5.30	2.60	0–10
Auditory phonological pattern recognition	8.55	1.44	0–12
Prosodic imitation score	4.09	0.88	0–10
Language self-efficacy	4.92	1.51	1–10

**Table 3:** Shows the correlations between NWS ADS reading and the variables under consideration.

		Melodic competence score											
Variables	NWS ADS	Singing happy birthday	MPT	MDT	MSA	BAT	Singing behavior during childhood	Musical engage-ment score	Passive music listening habits	Music relevance score	Auditory phonological pattern recognition	Prosodic lmitation score	Language self-efficacy
r	1	0.41**	0.44**	0.38**	0.16	0.21	0.27*	0.21	-0.07	0.03	0.16	0.04	-0.06

Sensitivity analyses showed that Singing Happy Birthday, MPT, and MDT consistently replaced one another as significant predictors of engaging fable presentation. Factor analysis confirmed these represent a single underlying construct (PCA loadings: MPT = 0.83, MDT = 0.85, Singing = 0.81), so a melodic competence composite was computed. *p < 0.05. **p < 0.01 (two-tailed).

Table 2 shows the correlations between NWS ADS reading and the musical variables under consideration, while Table 3 outlines the correlations between NWS Child-directed reading and the musical variables under consideration.

Child directed is the weakly significant correlation of the latter with BAT which assesses beat perception, i.e., a measure of rhythmic performance. Thus, it appears that this skill is apparently more important for CDS than for ADS (Table 4).

**Table 4:** Shows the correlations between NWS CDS reading and the variables under consideration.

		Melodic competence score											
Variables	NWS ADS	Singing happy birthday	MPT	MDT	MSA	BAT	Singing behavior during childhood	Musical engage-ment score	Passive music listening habits	Music relevance score	Auditory phonological pattern recognition	Prosodic imitation score	Language self-efficacy
r	1	0.43**	0.40**	0.41**	0.19	0.24*	0.26*	0.28*	-0.07	0.07	0.10	0.15	-0.10

*p < 0.05. **p < 0.01 (two-tailed).

### 3.3 Regressions

The variables used in this study are melodically dominant, as they primarily capture aspects of melodic perception and production. The correlation analyses revealed that the variables primarily reflecting melodic abilities (MDT, MPT, and singing) were highly interrelated. Sensitivity analyses conducted by alternately excluding each of the three variables from the regression models showed that these variables consistently replaced one another as significant predictors. Although the singing performance rating included range, rhythm, and voice quality, all dimensions reflect aspects of melodic execution and serve as indicators for melodic dominance in vocal performance. This pattern indicates that they tap into a common underlying melodic competence. To address this redundancy and reduce multicollinearity, a composite melodic score (melodic competence) was created. First, the individual melodic variables were standardized by transforming them into z-scores. These z-scores were then summed to form a single composite score representing the shared variance of melodic perception and production.

The melodic composite score, along with other variables that showed significant correlations (*p* < 0.05 change in F), were subsequently entered into the regression models. The dependent variables were the North Wind and the Sun (NWS) reading tasks.

#### 3.3.1 ADS reading

A multiple regression using the forced-entry method was performed. Results revealed that the melodic competence score (comprised of MPT, MDT, and singing) was the only significant predictor, explaining 25.3 percent of the variance in fable reading performance for the ADS reading. The corresponding Table 5 is presented below.

**Table 5:** Shows the regression model NWS ADS.

Multiple regression model explaining the variance in NWS ADS								
Model	*R*	*R* ^2^	*F* change	Sig. *F* change	*B*	*SE B*	*β*	*p*
Model 1	0.50	0.25	10.85	0.001				
Constant					5.11	0.30		<0.001
Melodic composite score					0.69	0.18	0.50	<0.001
Singing behavior during childhood					0.001	0.05	0.003	0.98

Dependent variable: NWS ADS.

#### 3.3.2 Child-directed reading

A multiple regression using the forced-entry method was also conducted for the CDS reading. Although more musical variables showed significant correlations with this reading condition compared to the ADS reading, the melodic competence score (comprised of MPT, MDT, and singing) remained the only significant predictor. This composite score uniquely explained 26.1 percent of the variance in fable reading performance for the child-directed reading. The corresponding Table 6 is presented below.

**Table 6:** Shows the regression model NWS CDS.

Multiple regression model explaining the variance in NWS CDS.								
Model	*R*	*R* ^2^	F change	Sig. F change	*B*	*SE B*	*β*	*p*
Model 1	0.52	0.27	5.64	0.001				
Constant					5.80	0.33		<0.001
Melodic composite score					0.66	0.18	0.57	<0.001
Singing behavior during childhood					−0.005	0.06	−0.02	0.93
BAT					−0.077	0.12	−0.09	0.50
Musical engagement score					0.015	0.22	0.01	0.95

Dependent variable: NWS CDS.

These results indicate that the melodic competence is the sole significant predictor in both reading conditions, explaining a comparable amount of variance in fable reading performance.

## 4 Discussion

This study addressed three main questions: First, whether musical skills relate to expressive reading of a fable to an intended audience. Second, we examined which specific musical variables showed the strongest associations with fable engagement across musicality dimensions, while also testing prosodic imitation and auditory phonological skills (requiring similar cognitive abilities as musical processing) alongside self-efficacy as competing predictors; third, we examined associations between musical measures and engaging fable presentation for CDS and ADS separately.

Correlational analyses revealed associations between expressive reading and melodic musicality variables, as well as childhood singing, in both adult- and CDS reading conditions. Rhythmic and musical engagement scores, however, showed correlations only in the CDS condition. The language measures – auditory phonological pattern recognition, prosodic imitation, and language self-efficacy – showed no significant associations with engaging fable presentation and therefore did not emerge as competing predictors. As a follow-up analysis, regression models were calculated. Sensitivity analyses revealed that the melodic variables (MDT, MPT, and singing) frequently replaced each other as significant predictors in the regression models, indicating that they tap into a common underlying melodic competence. This led to the creation of a composite melodic score, which emerged as the key predictor explaining individual differences in reading performance in both ADS and CDS.

### 4.1 Melodic variables and singing behavior during childhood

The melodic musicality variables, alongside childhood singing behavior, were the only two variables significantly associated with both ADS and CDS. Although childhood singing demonstrated bivariate correlations with ADS and CDS, regression analyses indicated that melodic ability emerged as the most robust and explanatory predictor. Research exploring the relationship between the multiple facets of musicality—including musical ability, singing behavior, and formal musical training—and expressive oral reading performance remains relatively scarce. Therefore, we address both singing behavior during childhood and melodic-related aspects from diverse perspectives to suggest that melodic ability may play a role in the expressive and skilled nature of oral reading performances, such as the reading of fables.

In language research, acquiring a language during childhood is widely recognized as a crucial predictor of achieving high proficiency later in life. Similarly, early engagement in musical activities, such as musical training and singing during childhood, is fundamental for developing musical expertise. Assessing childhood singing behavior and its relationship to expressive oral reading ability in adulthood allows for a cross-domain investigation. This cross-domain approach shows that childhood singing behavior is associated with both ADS and CDS speech. Previous research has demonstrated that childhood singing predicts not only singing ability but is also associated with oral foreign language pronunciation in adulthood. This relationship likely reflects the shared dependence of singing and oral language skills on vocal-motor and sensorimotor processes (Christiner et al., 2022a). Consequently, childhood singing may be associated with stronger vocal abilities throughout life, supporting the view that early vocal experiences are related to enduring aspects of expressive oral language skills. This broader category of expressive oral language may partly explain why we also observed a significant association between childhood singing behavior and expressive reading ability.

Building on the foundational role of vocal-motor mechanisms emphasized by childhood singing, research on expressive reading points to the important role of melodic features. Studies on expressive reading emphasize the pivotal role of melodic features in enhancing comprehension, sustaining attention, creating tension, and supporting memory, thereby increasing listener engagement. In the context of child studies, skilled readers have shown to possess larger pitch range and were also able to use pitch falls and rises more appropriately contrary to unskilled readers (Benjamin et al., 2013; Schwanenflugel et al., 2004). Expressive reading has been described as primarily related to pitch variations, including the extent of pitch movement, duration, and how these vary across sentences (Cowie et al., 2002). Another study on expressive reading suggests that pitch variation plays a crucial role in predicting reading comprehension skills, indicating that more attention should be given to pitch changes than to pause structures when examining the role of prosody in reading comprehension (Miller and Schwanenflugel, 2006). A study on adults has shown that adults with low literacy skills exhibit much flatter overall pitch variability compared to skilled readers. Although these readers do show the expected final pitch drop in declarative sentences, their intonation across the rest of the utterance remains nearly monotone (Binder et al., 2013). In our study, melodic ability was the only predictor that remained significant in the regression model for both the ADS- and CDS conditions, suggesting that melodic ability may contribute to individual differences in the expressiveness of reading performances. Given that our study involved adult participants that read a fable in their native languages, comprehension may be less critical here than in most of the studies cited earlier. Instead, attentional factors may play a more prominent role in our findings.

Playing with pitch variations in speech has been shown to be a very effective way of getting someone’s attention (Bailly et al., 2022). Research emphasizes that prosody primarily operates through melodic and pitch-based contrasts—such as high versus low pitch and rising versus falling contours—which not only mark stress patterns but are crucial in directing and sustaining the listener’s attention (Rodero, 2015). Supporting this, empirical findings demonstrate that speech containing pitch variations—particularly transitions from high to low pitch within a sentence—not only elicits greater listener engagement compared to speech with a homogeneous pitch range (Rodero et al., 2017) but are also perceived as the most effective and attractive by listeners (Rodero, 2022). It has been demonstrated that overuse of pitch variation in oral speech can be counterproductive, underscoring the necessity for its moderate and skillful application (Rodero et al., 2022). Our findings suggest that individuals with well-developed melodic sensitivity—typically associated with strong melodic musical abilities—are better equipped to modulate pitch variation effectively during reading, thereby enhancing its communicative impact for the intended audience.

Dynamic melodic variations in pitch are linked to enhanced expressiveness and to the perception of emotional tension, both of which are crucial for engaging listeners in speech and music. Larrouy-Maestri et al. (2025) summarize that dynamic melodic variations in fundamental frequency are critical acoustic features conveying emotional tension and engaging listeners during speech. In oral language processing, tonal abilities play a pivotal role, as they resemble musical skills in enhancing attentiveness and creating tension through deliberate pitch variations. Similar neural and perceptual mechanisms have been demonstrated in music research. Studies show that pitch variations function as important cues not only in speech prosody but also in musical melodies, where dynamic shifts in fundamental frequency capture attention and induce tension, engaging listeners’ cognitive processing (Zhang et al., 2022).

Melody seems to be a central feature associated with how a reading performance is perceived as attentive and how effectively it captures and maintains a listener’s attention throughout the narrative. Numerous studies on the overlap between language and music have discussed the critical role that melody plays in facilitating language memorization and aiding the recall of spoken utterances. For instance, new words are better remembered when they are sung (Ludke et al., 2014). Additionally, melody has been defined as a mnemonic device with which utterances are stored in long-term memory (Gordon et al., 2010). Previous research has demonstrated that individuals who perceive languages as exhibiting greater melodic qualities tend to achieve higher performance in pronunciation tasks and are found to be better intelligible, suggesting an important link between melodic perception and language production (Christiner et al., 2021). Moreover, melodic aptitude has been empirically linked to enhanced memorability of unintelligible utterance, indicating that the ability to perceive and produce pitch variations is associated with more efficient cognitive encoding and recall of speech (Christiner et al., 2023). Considering these findings in the context of our study, it is plausible to hypothesize that expressive reading performances perceived to be more melodic not only capture and sustain listener attention but are also more likely to be judged favorably, with higher assessments of skill and engagement by our raters.

### 4.2 CDS versus adult directed

We also wanted to investigate whether different musical measures predict expressive reading performance differently depending on whether the audience consists of children or adults. In contrast to ADS, CDS is characterized by higher pitch, greater pitch variability, exaggerated intonation, longer pauses, slower tempo, longer vowel duration which are thought to facilitate attention (Cristià, 2010; Fernald et al., 1989; McMullen and Saffran, 2004; Soderstrom et al., 2008). Therefore, we hypothesized that stronger associations between musical measures and expressive reading would be observed in the CDS condition compared to ADS. A robust relationship between expressive reading performance in the CDS context and melodic skills was identified. This finding is consistent with previous studies suggesting that CDS language is inherently more musical and songlike, primarily characterized by melodic variation (McMullen and Saffran, 2004; Nguyen et al., 2023). Contrary to our initial expectations, the expressive reading performances in CDS and ADS conditions differed only minimally. Notably, within the CDS condition, only the variable representing musical training—operationalized via the musical engagement score—and the CA-BAT measure, which assesses rhythmic ability, showed exclusive correlations with CDS reading performance. While the link between musical training and reading ability has been extensively debated (Partanen et al., 2022; Seither-Preisler et al., 2014), it may be more insightful to focus specifically on the rhythmic dimension. In our study there was a significant correlation between the CA-BAT measure in CDS but not ADS. CA-BAT measures perception of beats (i.e., rhythmic perception). Research has shown that rhythmic perception and production skills are closely related (e.g., Camici, 2025) and in general, CDS tends to be more rhythmic than ADS (e.g., Payne et al., 2015). Although the correlational associations align with previous research, rhythmic ability did not play a role when considering the results of the regression analyses (Camici, 2025; Payne et al., 2015).

While melodic musical skills emerged as the strongest predictors of individual differences in expressive reading performance in both conditions (ADS and CDS), passive listening habits, the relevance of music in daily life, and musical scene analysis (MSA) were not significantly associated with expressive reading in either condition. The two measures – passive listening habits and the personal relevance of music – pertain to passive forms of musical involvement and do not capture active engagement in music-making. Unlike melodic competence —which involve direct musical production and have shown robust associations with expressive reading—passive musical behaviors alone may be insufficient to facilitate improvements in expressive reading.

The MSA measure evaluates whether a specific musical target instrument is present within a complex musical composition or mix. This involves auditory scene analysis, which requires segregating overlapping sound sources—a process relying on auditory perception, selective attention, and working memory (Hake et al., 2024). In contrast, the melodic competence measures—consisting of the MDT, MPT, and singing—focus more on pitch discrimination and, in the case of singing, on vocal-motor control and expressive prosody. These fundamental differences likely explain why MSA does not correlate with expressive reading performance. The fixed ADS→CDS order prevents disambiguating speaking style from potential practice effects, which could be seen as a limitation of the study. Nonetheless, the convergent predictive models (*R*^2^ = 0.25/0.26; identical predictors) across readings support the reliability of our measures.

## 5 Conclusion

In this study, we investigated whether musical abilities, auditory skills, prosodic imitation, and self-efficacy are associated with rater-assessed expressive reading performance in CDS and ADS contexts. Correlational analyses revealed associations between expressive reading performance and melodic musicality variables (including childhood singing) across both conditions (ADS and CDS), while rhythmic/musical engagement correlated only in CDS, and language measures unexpectedly showed no associations. Among all predictors, regression analyses revealed that only a composite melodic ability score (MPT, MDT, singing) remained significant, explaining individual differences in rater-assessed expressive reading performance across both conditions. These findings highlight the consistent role of melodic musical abilities in expressive reading across CDS and ADS. Melodic competence correlated with expressive reading performance, potentially reflecting shared underlying processes such as attention, tension, and memory.

## Data Availability

The raw data supporting the conclusions of this article will be made available by the corresponding author upon reasonable request.
