# Supplementary material for: The influence of musicality on expressive fable reading: adult- and child-directed perspectives
Source: Front Psychol. 2026 Mar 16;17:1720324. doi: 10.3389/fpsyg.2026.1720324 (PMC13033758; doi:10.3389/fpsyg.2026.1720324)
Supplement: Supplementary file 1 [file Data_Sheet_1.pdf]

Supplementary Material

1 Intraclass correlation coefficients: Reading the Fable Nord Wind and the Sun

Table S1 and S2 display the intraclass correlation coefficients (ICCs) of the Fable reading Nord Wind and the Sun. Intraclass correlation coefficients (ICC) for average measures exceeding 0.7 are generally considered indicative of good interrater reliability. Table S1 reports the ICCs for the adult-directed fable reading, which was evaluated by 15 raters, whereas Table S2 presents the ICCs for the child-directed fable reading, rated by 14 raters. 15 raters were invited and participated in the evaluation; however, only 14 rated both the adult-directed and child-directed fable readings, while one rater provided ratings solely for the adult-directed fable reading. Both sets of ratings demonstrate good reliability, with ICC values surpassing the recommended threshold, thereby confirming a high level of agreement among raters.

Table S1. Intraclass correlation coefficients: Nord Wind and the Sun (adult-directed) 15 raters

		95% Confidence Interval		F Test with True Value 0			
	Intraclass Correlation	Lower Bound	Upper Bound	Value	df1	df2	Sig
Average Measures	.84	.74	.89	9.91	66	924	0,001

TableS2. Intraclass correlation coefficients: Nord Wind and the Sun (child-directed) 14 raters

		95% Confidence Interval		F Test with True Value 0			
	Intraclass Correlation	Lower Bound	Upper Bound	Value	df1	df2	Sig
Average Measures	.73	.60	.83	7.02	66	858	0,001

2 Intraclass correlation coefficients: Singing Happy Birthday

Tables S3 to S6 display the intraclass correlation coefficients (ICCs) of the rating criteria obtained for the first “Happy Birthday” singing. Table S5 shows the intraclass correlation coefficients (ICCs) obtained for the second Happy Birthday singing. These coefficients quantify the degree of agreement among raters, reflecting the reliability and consistency of the ratings assigned to participants’ performances.

Both sets of ratings demonstrate good reliability, with ICC values surpassing the recommended threshold, thereby confirming a high level of agreement among raters.

Table S3. Intraclass correlation coefficients: Singing Happy Birthday (melody)

	Intraclass Correlation	95% Confidence Interval		F Test with True Value 0			
		Lower Bound	Upper Bound	Value	df1	df2	Sig
Average Measures	.86	.76	.92	13.23	66	528	0,001

Table S4. Intraclass correlation coefficients: Singing Happy Birthday (quality of voice)

	Intraclass Correlation	95% Confidence Interval		F Test with True Value 0			
		Lower Bound	Upper Bound	Value	df1	df2	Sig
Average Measures	.80	.65	.88	11.42	66	528	0,001

Table S5. Intraclass correlation coefficients: Singing Happy Birthday (rhythm)

	Intraclass Correlation	95% Confidence Interval		F Test with True Value 0			
		Lower Bound	Upper Bound	Value	df1	df2	Sig
Average Measures	.70	.50	.82	7.41	66	528	0,001

Table S6. Intraclass correlation coefficients: Singing Happy Birthday (vocal repertoire)

		95% Confidence Interval		F Test with True Value 0			
	Intraclass Correlation	Lower Bound	Upper Bound	Value	df1	df2	Sig
Average Measures	.88	.79	.93	15.20	66	528	0,001

3 Scales for Singing Behavior During Childhood, Music Relevance, and Passive Music Listening Habits

The following sections present the individual items (translated from German into English) and reliability analyses for the multi-item scale constructs assessing Singing Behavior During Childhood, Music Relevance, and Passive Music Listening Habits.

3.1 Singing behaviour during childhood

The multi-item scale assessing singing behaviour during childhood comprised ten items, each rated on an 11-point Likert scale ranging from 0 (strongly disagree/not at all) to 10 (strongly agree/completely). The internal consistency of singing behaviour during childhood was assessed using Cronbach's alpha, yielding a value of $\alpha = 0.92$, indicating acceptable reliability.

Examples of items include:

a. As a child I enthusiastically joined in with the singing at church and similar events whenever the possibility arose.
b. As a child I was encouraged to sing by my caretakers and we sang together on a weekly basis even if there were no special events.
c. As a child I enjoyed singing in a choir, with friends, at Christmas, birthdays, or at similar occasions.
d. As a child I sang very often since I wanted to become a musician or singer.
e. As a child I used to sing whenever I could such as in the bathroom, in the car, when I played with friends
f. As a child I liked being a member of our school choir, or would have liked being a member or a school choir.
g. As a child I enjoyed singing a song that had been played to me (e.g., in the radio).
h. As a child I used to sing more often than my friends.
i. In my childhood singing played a major role
j. As a child my parents did not sing a lot with me.

3.2 Musical relevance

The multi-item scale assessing musical relevance comprised five items, each rated on an 11-point Likert scale ranging from 0 (strongly disagree/not at all) to 10 (strongly agree/completely). The internal consistency of musical relevance was assessed using Cronbach's alpha, yielding a value of α = 0.78, indicating acceptable reliability.
Examples of items include:

a. I would not describe myself as a musician.
b. I grew up with a lot of music.
c. When I was a child, my parents listened to music all the time.
d. Music has always been an important part of my life.
e. I listened to someone play an instrument at least once a week as a child.

3.3 Passively Listening to Music

The multi-item scale assessing passive music listening comprised four items, each rated on an 11-point Likert scale ranging from 0 (strongly disagree/not at all) to 10 (strongly agree/completely). The internal consistency of musical relevance was assessed using Cronbach's alpha, yielding a value of α = 0.74, indicating acceptable reliability.
Examples of items include:

a. When I listen to music while I study, it's much easier for me to keep going.
b. I believe that I learn things more easily when I listen to music.
c. I hear music in the background all the time.
d. I listen to more than 5 hours of music a day (e.g. radio, Spotify, or similar).

4. Language Self-Efficacy

The 7-item scale assessing task-specific language abilities comprised seven items, directly referencing tasks performed in the study, each rated on a visual analogue scale ranging from 0 (strongly disagree) to 10 (strongly agree). The internal consistency was assessed using Cronbach's α = .74, indicating acceptable reliability. The statements are provided below:

a. The speech listening tasks which I performed before are easy.
b. Imitating languages such as pronunciation exercises in foreign languages I do very well.
c. I would estimate my expressive reading ability as very good.
d. I have very good potential to learn languages.
e. I have no difficulties when I am asked to solve language tasks.
f. I am a language talent and learn languages very quickly.
g. I'm good at storytelling.

5. Intraclass correlation coefficients: Prosodic imitation in Tagalog and Mandarin

Tables S7 to S18 display the intraclass correlation coefficients (ICCs) of the Tagalog and Mandarin ratings for the six sentences in each language. Intraclass correlation coefficients (ICCs) were initially calculated using the full sample to assess the reliability of participant performance ratings.

Table S7. Intraclass correlation coefficients: Tagalog sentence

		95% Confidence Interval		F Test with True Value 0			
	Intraclass Correlation	Lower Bound	Upper Bound	Value	df1	df2	Sig
Average Measures	.90	.86	.94	13.48	63	945	0,001

Table S8. Intraclass correlation coefficients: Tagalog sentence

		95% Confidence Interval		F Test with True Value 0			
	Intraclass Correlation	Lower Bound	Upper Bound	Value	df1	df2	Sig
Average Measures	.94	.92	.97	23,03	63	945	0,001

Table S9. Intraclass correlation coefficients: Tagalog sentence

		95% Confidence Interval		F Test with True Value 0			
	Intraclass Correlation	Lower Bound	Upper Bound	Value	df1	df2	Sig

	Intraclass Correlation	Lower Bound	Upper Bound	Value	df1	df2	Sig
Average Measures	.93	.89	.95	20,07	65	975	0,001

Table S10. Intraclass correlation coefficients: Tagalog sentence

		95% Confidence Interval		F Test with True Value 0			
	Intraclass Correlation	Lower Bound	Upper Bound	Value	df1	df2	Sig
Average Measures	.95	.92	.96	23,06	63	945	0,001

Table S11. Intraclass correlation coefficients: Tagalog sentence

		95% Confidence Interval		F Test with True Value 0			
	Intraclass Correlation	Lower Bound	Upper Bound	Value	df1	df2	Sig
Average Measures	.92	.89	.95	18,16	65	975	0,001

Table S12. Intraclass correlation coefficients: Tagalog sentence

		95% Confidence Interval		F Test with True Value 0			
	Intraclass Correlation	Lower Bound	Upper Bound	Value	df1	df2	Sig
Average Measures	.90	.86	.94	14,43	65	975	0,001

Table S13. Intraclass correlation coefficients: Mandarin sentence

		95% Confidence Interval		F Test with True Value 0			
	Intraclass Correlation	Lower Bound	Upper Bound	Value	df1	df2	Sig
Average Measures	.97	.96	.98	49,87	63	2520	0,001

Table S14. Intraclass correlation coefficients: Mandarin sentence

		95% Confidence Interval		F Test with True Value 0			
	Intraclass Correlation	Lower Bound	Upper Bound	Value	df1	df2	Sig
Average Measures	.98	.97	.98	62,42	62	2480	0,001

Table S15. Intraclass correlation coefficients: Mandarin sentence

		95% Confidence Interval		F Test with True Value 0			
	Intraclass Correlation	Lower Bound	Upper Bound	Value	df1	df2	Sig
Average Measures	.98	.96	.98	59,55	65	2535	0,001

Table S16. Intraclass correlation coefficients: Mandarin sentence

	Intraclass Correlation	95% Confidence Interval		F Test with True Value 0			
		Lower Bound	Upper Bound	Value	df1	df2	Sig
Average Measures	.98	.96	.98	55,65	64	2496	0,001

Table S17. Intraclass correlation coefficients: Mandarin sentence

	Intraclass Correlation	95% Confidence Interval		F Test with True Value 0			
		Lower Bound	Upper Bound	Value	df1	df2	Sig
Average Measures	.98	.97	.98	75,30	63	2457	0,001

Table S18. Intraclass correlation coefficients: Mandarin sentence

	Intraclass Correlation	95% Confidence Interval		F Test with True Value 0			
		Lower Bound	Upper Bound	Value	df1	df2	Sig
Average Measures	.96	.95	.98	44,72	64	2496	0,001

6. Auditory phonological pattern recognition stimuli in Mandarin

For the development of this test, the stimuli for the auditory phonological pattern recognition measure were segmented into word and short phrase sequences of three to eleven syllables, capturing

authentic phonological and prosodic characteristics. Segment boundaries were determined by natural pauses in the speech stream. In the case of Mandarin, 24 samples were derived from each speaker.

Each trial begins with a reference string stimulus comprising segments that embody realistic phonological and prosodic features. This is followed by a response stimulus featuring one to three segments, varying by experimental condition. For responses with multiple segments, all had to appear somewhere in the reference string to be scored as correct, regardless of their sequential order. For example, a two-segment response was deemed accurate if both elements were present in the original string, independent of their positions. Every task pair (reference string and response) exclusively utilized material from the same speaker.

Examples of Mandarin samples from Speaker 1 are provided below. Table S19 specifies the phrases drawn from the fable "The North Wind and the Sun" in Mandarin.

Samples in Mandarin
yǒu-yī-cì běi-fēng
hé tài-yáng zhèng-zài zhēng-lùn shéi
bǐ-jiào qiáng
tāmen zhènghǎo kàndào
yǒugè lùrén zǒuguò
nàgèrén chuānzhè yījiàn dǒupéng
tāmen jiù juédìng
shéi kěyǐ ràng lùrén
tuōdiào nàjiàn dǒupéng
jiùsuàn shéi bǐjiào lìhài
yúshì běifēng jiù
pīnmìngdì chuī
méi xiǎng dào， tā chuīdé yù lìhài
lùrén jiù yùshì yòng
dǒupéng bāojǐn zìjǐ.
zuìhòu, běifēng méi bànfǎle
zhǐhǎo fàngqì.
jiēzhe， tàiyáng chūlái wēnnuǎndi
zhàoyàole yīxià
lùrén jiù lìkè bǎ dǒupéng tuōdiàole
yúshì, běifēng zhǐhǎo rènshū
chéngrèn tàiyáng
bǐjiào lìhài

7. Exploratory stepwise multiple regression analyses

Exploratory stepwise multiple regression analyses were conducted to screen predictors prior to confirmatory forced-entry models reported in the main text. Variables were entered based on $p < .05$ change in F criterion. Results identified the Melodic composite score as the sole significant predictor in both models, explaining 25% (ADS) and 26% (CDS) of variance. These findings were fully replicated by confirmatory forced-entry analyses (Tables S20–S21).

Table S20 shows the stepwise regression model ADS.

Multiple regression model explaining the variance in NWS ADS

	R	R^2	F Change	Sig. F Change	*B*	*SE B*	*β*	*p*
Model 1	.50	.25	22.03	0.000				
Constant					5.11	0.12		
Melodic composite score					0.70	0.15	.50	< .001

Dependent variable: NWS ADS

Table S21 shows the stepwise regression model CDS.

Multiple regression model explaining the variance in NWS CDS

	R	R^2	F Change	Sig. F Change	*B*	*SE B*	*β*	*p*
Model 1	.51	.26	22.98	0.000				
Constant					5.80	0.10		
Melodic composite score					0.59	0.12	.51	< .001

Dependent variable: NWS Child-directed
